# Associations between Longitudinal Maternal and Cord Blood Vitamin D Status and Child Growth Trajectories Up to 4 Years of Age

**DOI:** 10.3390/nu16152410

**Published:** 2024-07-25

**Authors:** Chen Chen, Chunyan Zhou, Jun Zhang, Ying Tian, Xirui Wang, Xianting Jiao, Yue Zhang, Xiaodan Yu

**Affiliations:** 1 Department of Developmental and Behavioral Pediatrics, Shanghai Children’s Medical Center, School of Medicine, Shanghai Jiao Tong University, Shanghai 200127, China; 2 Translational Medicine Institute, Shanghai Children’s Medical Center, School of Medicine, Shanghai Jiao Tong University, Shanghai 200127, China; 3 MOE-Shanghai Key Laboratory of Children’s Environmental Health, Xinhua Hospital, School of Medicine, Shanghai Jiao Tong University, Shanghai 200092, China; 4 Department of Pediatric Cardiology, Xinhua Hospital, School of Medicine, Shanghai Jiao Tong University, Shanghai 200092, China

**Keywords:** maternal vitamin D, cord blood vitamin D, growth trajectory, group-based trajectory model (GBTM), children

## Abstract

The current study aimed to explore the combined and individual effects of vitamin D (VitD) status in three trimesters during pregnancy and cord blood (CB) on child growth trajectories from birth to 4 years of age. Pregnant women (n = 1100) were recruited between 2013 and 2016 in the Shanghai Birth Cohort (SBC) Study. A total of 959 mother–child dyads were included. VitD status was measured by LC-MS/MS at three trimesters (T1, T2, T3) and CB. Children’s weight, length/height, and head circumference were assessed at birth, 42 days, 6, 12, 24 months, and 4 years of age, and standardized into z-scores [weight-for-age z-score (WAZ), length-for-age z-score (LAZ), head circumference-for-age z-score (HCZ) and weight-for-length z-score (WLZ)]. Using the group-based trajectory model (GBTM), the trajectories of the four growth parameters were categorized into discrete groups. The generalized estimating equation (GEE) was employed to analyze the mixed effect of 25(OH)D throughout pregnancy on growth trajectories. The association between 25(OH)D status and each growth trajectory group was examined by multivariable logistic regression. Each 10 ng/mL increase in 25(OH) throughout three trimesters was not associated with four anthropometric parameters. Each 10 ng/mL increase in VitD in T3 was associated with a lower risk in the WAZ high-increasing trajectory (aOR: 0.75; 95% CI: 0.62, 0.91; *p* < 0.01). Each 10 ng/mL increase in VitD in CB was associated with a lower risk in the WAZ high-increasing trajectory (aOR: 0.57; 95% CI: 0.43, 0.76; *p* < 0.01). No significant association was found between maternal or CB VitD and LAZ or HCZ. Three trimesters’ VitD throughout pregnancy had no persistent effect on the offspring’s growth trajectory. However, higher VitD status in the third trimester and CB related to a lower risk of high-increasing WAZ from birth to 4 years of age. Elevated VitD levels in late pregnancy and cord blood may protect against continuous early-life weight growth at high levels.

## 1. Introduction

Vitamin D (VitD) is well-known as a fat-soluble steroid hormone for its crucial effects on maintaining extra-skeletal health [1,2]. VitD plays a crucial role in growth and development due to its substantial contribution to cellular growth, differentiation, and maturation processes [3]. A deficiency in vitamin D can potentially disrupt calcium equilibrium, bone mineralization, and fat metabolism, which are all integral elements for fetal growth and development [4]. The impact of maternal or cord blood (CB) VitD on offspring body size and obesity in early childhood and later life has gained growing attention in recent years [2,5]. The association between maternal or CB VitD levels and childhood growth remains unclear and differs across various developmental stages [6,7]. Previous studies have mainly suggested that maternal VitD status was negatively associated with the risk of low birth weight [6], elevated whole body fat mass in neonates [8], increased body mass index (BMI) z-score, and central obesity in school-aged children [9]. Nevertheless, conflicting evidence is still emerging. The Danish birth cohort [7] and the Chinese prospective study [10] reported no significant association in children from birth to 3 years of age. Beyond indicators of adiposity, the influence of maternal or cord blood Vitamin D levels on other growth parameters, such as length and head circumference, is seldom explored.

Prior studies focused on the relationship between maternal or CB VitD concentration and one-time or several times static anthropometric measures rather than constructing dynamic growth trajectory. Tracking and identifying unfavorable growth trajectories in early childhood might capture the physical health problems in later life [11,12,13]. Growing evidence strongly suggests that rapid and increased growth patterns during early childhood can potentially forecast obesity in later childhood and adulthood [14,15]. Adipogenesis, starting in utero and accelerating during the neonatal period, is currently acknowledged as a crucial factor influencing the rapid increase in global obesity or type 2 diabetes mellitus prevalence [16]. Therefore, linking modifiable early risk factors during the fetal period and offspring growth trajectories may offer new perspectives for designing preventive strategies [15].

The impact of VitD status across all three trimesters on children’s growth trajectory has not been previously reported. Earlier studies examined VitD status during individual trimesters, such as the first (T1) [17], second (T2) [18], and third trimester (T3) [19], instead of evaluating all three trimesters in detail. Only one cohort collected VitD status four times and explored their relation to neonatal size outcomes, discovering that the associations were dependent on the timing of VitD measurements [20]. However, further investigation is needed to understand the interaction between VitD levels in all three trimesters, as well as the actual effect of a single trimester and the ongoing influence of maternal VitD.

The current study aimed to characterize distinct growth trajectories of children from birth to 4 years of age and subsequently examine the combined effect and separate effect of three trimesters’ maternal VitD and CB VitD on identified growth trajectories, by utilizing the Shanghai Birth Cohort (SBC), which includes three trimesters’ VitD data and regular anthropometry follow-up visits.

## 2. Materials and Methods

### 2.1. Study Participants

**Figure 1:**
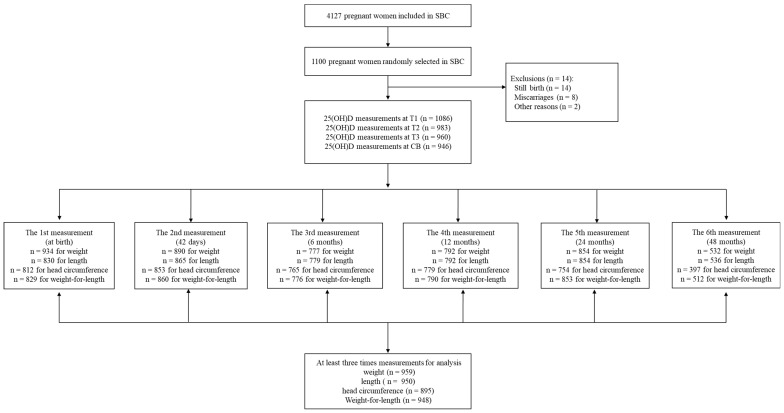
Flowchart of the study participants in Shanghai Birth Cohort (SBC), Shanghai, China, recruited from 2013 to 2016. T1, early pregnancy; T2, middle pregnancy; T3, late pregnancy; and CB, cord blood.

Participants were derived from the SBC study and the cohort’s characteristics were previously detailed [21]. The criteria for inclusion in our study required completed 25(OH)D measurements during early pregnancy (<16 weeks, T1), mid-pregnancy (24–28 weeks, T2), late pregnancy (32–36 weeks, T3), and CB. Additionally, child follow-up physical measurements were needed at least three times from birth to 4 years of age (at birth, 42 days, 6, 12, 24 months, and 4 years of age). Exclusion criteria were miscarriage, stillbirth, and migration out of the municipality. Figure 1 illustrates the flowchart of participants eligible for analysis. A total of 1100 out of the 4127 pregnant women were randomly chosen. Of them, 1086 at T1, 983 at T2, 960 at T3, 946 in CB finished 25(OH)D measurement. Children with at least three times four growth indicators from birth to 4 years of age were included in the study population. Finally, there were 959, 950, 859, and 948 participants included for weight, length, head circumference, and weight-for-length statistical analysis, respectively.

All the participants signed written informed consent. The research was approved by the Ethics Committees of Xinhua Hospital affiliated with Shanghai Jiao Tong University School of Medicine (XHEC-C-3-001-3). The Strengthening the Reporting of Observational Studies in Epidemiology (STROBE) reporting guideline was adhered to in this study. (https://www.strobe-statement.org/checklists/) (accessed on 1 July 2018).

### 2.2. 25-Hydroxyvitamin D Measurements

Blood samples gathered during the three trimesters of pregnancy and cord blood were separated via centrifugation and stored in vials at a temperature of −20 °C. Sensitive liquid chromatography–tandem mass spectrometry (LC-MS/MS) (Agilent Technologies Inc., Santa Clara, CA, USA) was used to analyze the serum samples (100 μL) in accordance with the protocol described in our current investigation [22]. For 25(OH)D2 and 25(OH)D3, the sensitivity level of the LC-MS/MS test was 0.05 ng/mL and 0.10 ng/mL, respectively. For every set of samples, at least two quality control measures were taken. The sum of 25(OH)D_2_ and 25 (OH)D_3_ was used to compute the overall 25(OH)D concentration.

We referred to the Institution of Medicine (IOM) [23] and the Endocrine Society for reference values of VitD in pregnant women [24]. We classified the maternal VitD status as deficiency (< 20 ng/mL), insufficiency (≥20 ng/mL, <30 ng/mL), and sufficiency (≥30 ng/mL). As for CB VitD, based on the widely recognized guidelines for the general public and the results of current research on newborns [24,25], CB 25(OH)D concentration below 12 ng/mL, ≥12 ng/mL, <20 ng/mL, and ≥20 ng/mL is indicated as deficient, insufficient, and sufficient, respectively. The criteria we referenced are consistent with those in our previous study [26]. Average VitD status during three trimesters was calculated as the mean 25(OH)D value of three VitD measured time points.

### 2.3. Anthropometric Measurements

Weight, length/height, and head circumference were measured when children were at birth, 42 days, 6, 12, 24 months, and 4 years of age by trained nurses who were blinded to the 25(OH)D measurement. The precision of the weight was 0.1 kg, while the head circumference and length/height were both within 0.1 cm. Using the World Health Organization (WHO) Anthro Packages, the raw data on weight, length/height, and head circumference were transformed into weight-for-age (WAZ), length-for-age (LAZ), head circumference-for-age (HCZ), and weight-for-length z-score (WLZ) [27].

### 2.4. Covariates

All participants were interviewed by the trained research assistant. A set of questionnaires disseminated to the mother during pregnancy and follow-up visits were collected, including maternal age at delivery, maternal prepregnancy body mass index (BMI), maternal educational level, prepregnancy weight, postpartum weight, and history of gestational diabetes mellitus (GDM). Additional significant factors, such as child sex, birth type, and delivery mode, were extracted from hospital medical records. The postpartum weight at delivery less the prepregnancy weight was used to calculate gestational weight gain.

### 2.5. Statistical Analyses

The group-based trajectory model (GBTM) was used to construct growth trajectories from birth to 4 years of age. GBTM is mainly used to explore the heterogeneity in the longitudinal data and there are several potential subgroups with different developmental trajectories. All participants are assumed to be from the same population; however, they are divided into different groups based on their differences in growth [28].

Following model selection suggestions from previous studies [29], we identified the growth trajectories over time. The selection of the optimal number of trajectory groups and the power of the polynomial are decided according to the Bayesian information criteria (BIC). When performing the fitting, the fitting is usually started from the lower power or fewer number of trajectory groups, and lower power or fewer number of trajectory groups are defined as the null model, and models with increasing powers or trajectory groups are called the complex model. The fitting effect of the two models is judged by 2∆BIC [2∆BIC = 2 (BIC _complex model_ − BIC _null model_)]. If 2∆BIC < 2, the null model is accepted; if 2∆BIC ≥ 2, the complex model is accepted. We developed models containing varying numbers of trajectory groups (ranging from one to five) using a quadratic form for all trajectories, as shown in Supplementary Table S1. We then calculated the corresponding BICs. The quadratic function employed at this stage aided in identifying the ideal number of groups. We chose a 3-4-3-3 group-based trajectory model to describe WAZ, LAZ, HCZ, and WLZ trajectories. A higher BIC signifies a superior model fit, with 2∆BIC < 2 indicating insufficient evidence against the null model, which was solely used for determining the optimal group number. Subsequently, we identified the best trajectory shapes that accurately represented the observed trajectories. We tested linear, quadratic, cubic, quartic, and quintic functions for this purpose (Supplementary Table S2). Ultimately, the cubic function was chosen to characterize the trajectory shape of WAZ, LAZ, and LAZ, while the linear function was used for HCZ. Upon determining the ideal number of trajectory groups and the appropriate trajectory shape, we visually depicted the trajectories.

Next, we described the distribution of VitD status and maternal–child characteristics selected for analysis according to the four growth measurements. The generalized estimating equation (GEE) was employed to investigate the mixed effect of maternal VitD status throughout three trimesters on growth trajectories. Then, multivariable logistic regression was employed to explore the association of maternal VitD status (both each 10 ng/mL change and categorization) in three trimesters, CB and average VitD during pregnancy with growth trajectory outcomes after adjustment for confounders. A priori selection or univariate analysis revealed potential confounders such as child sex, delivery mode, gestational weight increase, maternal age, maternal education level, and pre-pregnancy BMI. We performed stratified analyses to assess the potential impact of child sex and maternal age (categorized as <30 or ≥30 years) on offspring growth, as well as to determine if these factors modify the associations between VitD status and anthropometric outcomes.

The GBTM was executed utilizing PROC TRAJ in SAS, while additional analyses were carried out in Empower (R) (www.empowerstats.com, X&Y solutions, Inc., Boston, MA, USA) and R 3.5.1 (http://www.R-project.org) (accessed on 31 December 2020). All *p* values were derived from a two-tailed test, and a *p* value of < 0.05 signified a statistically significant difference. Moreover, the *p* value was adjusted with Bonferroni correction in multiple tests.

## 3. Results

Table 1 displays the VitD distribution and maternal–child characteristics. The mean ± SD of VitD concentration in T1, T2, T3, and CB is 26.16 ± 10.20 ng/mL, 31.93 ± 11.37 ng/mL, 35.75 ± 13.33 ng/mL, and 19.87 ± 9.07 ng/mL, respectively. Most of the recruited women are aged between 25 to 30 years, with a college degree or above (70.36%), and have normal prepregnancy BMI (91.27%), and no gestational diabetes mellitus (GDM) history (84.34%).

**Figure 2:**
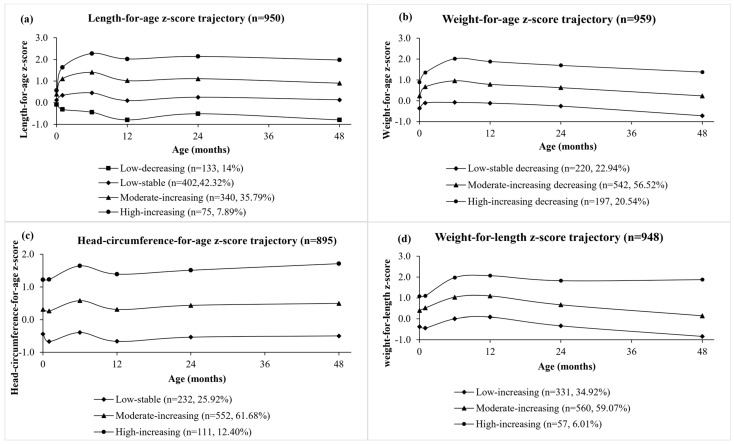
Growth trajectories for length-for-age z-score (**a**), weight-for-age z-score (**b**), head circumference-for-age z-score (**c**), and weight-for-length z-score (**d**) from birth to 4 years of age.

The original anthropometric measurement values and their corresponding z-scores can be found in Supplementary Table S3. The GBTM revealed distinct trajectory groups for the four growth parameters, with the trajectories for each group illustrated in Figure 2. The trajectories were named based on their initial values (high, moderate, low) and following patterns (increasing, stable, decreasing). WAZ was categorized into three groups: low-stable (n = 220, 22.94%), moderate-increasing (n = 543, 56.52%), and high-increasing (n = 197, 20.54%). LAZ was categorized into four groups: low-decreasing (n = 133, 14.00%), low-stable (n = 402, 42.32%), moderate-increasing (n = 340, 35.79%), and high-increasing (n = 75, 7.89). HCZ was categorized into three groups: low-stable (n = 232, 25.92%), moderate-increasing (n = 552, 61.68%), and high-increasing (n = 111, 12.40%). WLZ was categorized into three groups: low-increasing (n = 331, 34.92%), moderate-increasing (n = 560, 59.07%), and high-increasing (n = 57, 6.01%) (Supplementary Table S4).

Table 2 presents the persistent effect of maternal VitD status in three trimesters on growth trajectory groups. No significant mixed effect of maternal VitD on all growth parameters was observed. Also, no notable relationship was identified between the average VitD levels across the three trimesters and the four growth parameters (Supplementary Table S5). Table 3 shows the associations between maternal VitD at each trimester and CB and growth trajectories. After adjusting maternal age, maternal education, pre-pregnancy BMI, gestational weight gain, delivery mode, and child sex, each 10 ng/mL VitD increase in the third trimester was associated with a lower risk of WAZ high-increasing trajectory (aOR: 0.75; 95% CI: 0.62, 0.91; *p* < 0.01). Each 10 ng/mL VitD increase in CB was associated with a lower risk of WAZ high-increasing trajectory (aOR: 0.57; 95% CI: 0.43, 0.76; *p* < 0.01). After categorizing the VitD status into deficient and insufficient, and sufficient groups (Supplementary Table S6), compared with T3 sufficient group (≥30 ng/mL), VitD < 30 ng/mL in T3 significantly increased the risk of WAZ high-increasing trajectory (aOR: 2.04; 95% CI: 1.27, 3.26; *p* < 0.01), WLZ high-increasing trajectory (aOR: 2.97; 95% CI: 1.51, 5.82; *p* < 0.01). When compared to the CB VitD status ≥20 ng/mL group, CB VitD levels <20 ng/mL were linked to a higher risk of the WAZ high-increasing trajectory (aOR: 2.58; 95% CI: 1.60, 2.02; *p* < 0.01), the LAZ moderate-increasing trajectory (aOR: 1.56; 95% CI: 1.13, 2.14; *p* < 0.01), and the WLZ moderate-increasing trajectory (aOR: 1.50; 95% CI: 1.12, 4.16; *p* = 0.02). There was no notable correlation between VitD levels in T3 or CB and HCZ. Additionally, there was no significant relationship between 25(OH)D levels in T1, T2, and the four growth parameter trajectories.

The gender stratification analysis is presented in Supplementary Table S7. For males, each 10 ng/mL VitD increase in T3 significantly related to reduced risk of the WAZ high-increasing group (aOR: 0.67; 95% CI: 0.50, 0.88; *p* = 0.01); each 10 ng/mL VitD increase in CB significantly related to reduced risk of the WAZ moderate-increasing group (aOR: 0.76; 95% CI: 0.60, 0.96; *p* = 0.04) and WAZ high-increasing group (aOR: 0.46; 95% CI: 0.30, 0.70; *p* < 0.01). Supplementary Table S8 demonstrates the analysis stratified by maternal age. In the maternal age < 30 group, each VitD 10 ng/mL increase in CB was associated with a lower risk of the WAZ high-increasing group (aOR: 0.61; 95% CI: 0.42, 0.89; *p* = 0.02). In the maternal age ≥ 30 group, each VitD increase in T3 (aOR: 0.63; 95% CI: 0.44, 0.89; *p* < 0.01) and CB (aOR: 0.54; 95% CI: 0.34, 0.83; *p* < 0.01) was associated with a lower risk of being in the WAZ high-increasing group.

## 4. Discussion

To our knowledge, this is the first study to investigate the combined and individual effect of VitD status at three trimesters during pregnancy and CB on postnatal growth parameter trajectories in the first 4 years of life. By using the novel GBTM model, we characterized postnatal WAZ, LAZ, HCZ, and WLZ from birth to 4 years of age into distinct trajectories. VitD status throughout the three trimesters was not significantly associated with postnatal growth trajectories. However, we found higher VitD status in the third trimester and CB was significantly related to a lower risk of the WAZ high-increasing trajectory.

Our study found lower VitD status in the third trimester and CB was significantly related to the WAZ high-increasing trajectory from birth to 4 years of age. To our knowledge, only two longitudinal studies examined how VitD concentration during pregnancy may influence postnatal BMI trajectories [10,30]. A Swedish cohort study categorized infant BMI growth patterns from birth to 2 years into two groups: a stable normal and a stable high class, using growth mixture modeling (GMM) [30]. Their findings indicated that maternal 25(OH)D levels at or below 30 ng/mL might be linked to the stable high class of BMI growth trajectory. Another study was conducted in China, which identified stable moderate and early transient BMI-Z groups from birth to 2 years of age with a latent class growth mixture (LCGM) model, however, this study produced null associations [10]. The two studies did not thoroughly investigate other growth factors like weight, length, and head circumference, and concentrated on the vague periods of maternal VitD status and BMI trajectory during the first 2 years of life. By using a different and novel trajectory model, our study provided further evidence by showing the association between maternal VitD concentration at each trimester as well as throughout the entire pregnancy, and four growth parameters trajectories from birth to 4 years of age. We ascertained that while there was no lasting impact of VitD status from all three trimesters on growth patterns, VitD status in late pregnancy and CB is crucial in regulating weight trajectories.

Only two existing studies have reported the associations between the repeatedly measured maternal VitD status and postnatal growth, but none of them examined the persistent effect of VitD throughout pregnancy or the interactions of maternal VitD in different trimesters. Our study first reported that VitD throughout three trimesters has no persistent effect on offspring growth trajectories. The NICHD Fetal Growth Studies–Singleton’s cohort collected four times VitD concentrations during pregnancy to investigate the relation to neonatal anthropometry and observed the associations between maternal VitD, and the sum of skinfolds and neonatal birth-weight z-score were specific to gestational weeks of 25(OH)D assessed, pointing out that VitD plays its role in neonatal anthropometry in each trimester [20]. The Odense Child Cohort collected blood samples both in CB, early and late pregnancy, however, no association between 25(OH)D concentrations and body fat or adiposity was found in children at 3 years of age [7]. The two above-mentioned studies performed the point-to-point analysis of maternal VitD and physical growth over time instead of analyzing the continuous effect of maternal VitD and the dynamic growth pattern. Other explanations include the study population’s variability, mothers’ life habits [31], the interaction impact between VitD and other variables, as well as other confounding factors, such as the effect of VitD supplements during pregnancy and after birth. Further studies are needed to clarify the lasting impact and the interplay of maternal VitD on the growth of offspring and to pinpoint the crucial period for introducing VitD interventions while taking into account a wider range of contributing factors.

We identified VitD in the third trimester and CB was negatively associated with postnatal weight pattern, as each 10 ng/mL increase in VitD at two stages considerably lowered the risk of the WAZ high-increasing growth trajectory. Maternal VitD at late pregnancy had the strongest correlation with VitD status in CB [26,32], determining the baseline VitD status for postnatal growth; therefore, it is reasonable to find that VitD in CB also has a great effect on postnatal weight growth. The physiological reality that muscle and bone development start in early pregnancy, while the fetus primarily gains weight through fat mass accumulation in late pregnancy, could partially contribute to this outcome [33]. So, the nutrients or nutrition can be fully utilized in late pregnancy to promote fat increase, leading to weight gain. Another possible explanation for that might be the modifiable effect of maternal dietary intake during pregnancy because carbohydrate intake at late but not early gestation was positively associated with fat mass at birth and after birth [34,35]. In our study, the maternal intake of food components during pregnancy was not able to be calculated in detail, which limited our study to estimate the effect of the interaction between other elements in the diet and VitD on overweight or obesity in the offspring. VitD in the third trimester is particularly important for children with mothers above 30 years of age, while CB VitD is equally essential for children’s WAZ trajectory irrespective of their mother’s age.

Though no significant association was found between maternal or CB VitD and LAZ, HCZ. The differential sex effect of VitD status on HCZ was observed in this study. Particularly for males, higher CB VitD status is associated with a lower risk of high-increasing HCZ trajectory. Our finding aligns with a prior study that demonstrated a relation between higher CB 25(OH)D levels and reduced head circumference at 6 and 12 months [17]. Conflicting results have been reported, indicating that CB VitD levels did not show any association with infant length or head circumference at any stage between 3 months and 24 months [36] or 4 years of age [37], while other reported daily maternal VitD supplementation significantly increased head circumference [38]. Different ages, the prevalence of VitD deficiency, and racial differences may explain the above-mixed findings. Further study on this issue is needed. CB VitD is negatively associated with moderate-increasing LAZ trajectory in children with mothers aged below 30. That means appropriate VitD concentration could prevent excessive as well as slow length/height growth. For infants and toddlers, it is primarily reported that maternal and CB VitD status was negatively correlated with LAZ [17,36,39].

Our study pointed out the importance of vitamin D status in late pregnancy and cord blood and its associations with offspring growth and weight gain. Considering the low vitamin D supplement rate in pregnant women, which was 8.97 % in Chinese National Nutrition Surveillance 2010–2012 and 2015–2017 [40], this research highlights the significance of maintaining adequate vitamin D status during the third trimester and encourages the healthcare professionals to raise awareness among expectant mothers about the necessity of consistent vitamin D supplementation as advised by the Chinese Nutrition Medicine Association. Furthermore, this study underscores the value of monitoring growth patterns, which can help predict weight gain trends and provide pediatricians with essential information for offering feeding guidance or other recommendations to prevent the onset of obesity.

A major strength of our study is that it is the most comprehensive study to date to investigate the associations of maternal VitD status at three trimesters and CB and postnatal growth trajectories. In addition, we applied GBTM to characterize postnatal growth trajectories from birth to 4 years old, which allows for identifying heterogeneous subgroups and compensates for studies of consecutive single time points. Another novelty of the study is the well-designed prospective multi-center study with a representative population sample and extensively collected covariates, including maternal age, maternal education, prepregnancy BMI, etc.

There are several limitations to be considered. First, the participant’s inclusion criterion that all should have at least measurements of weight and length/height might induced potential selection bias since we excluded participants with less than three times measurements. Second, although we have examined confounding factors as comprehensively as possible, residual and unknown confounding cannot be ruled out, such as maternal diet and lifestyle habits during pregnancy. Third, due to the possibility of misclassification of GBTM, as well as other trajectory analysis methods, trajectory explanation to each individual should be cautious. Although these results are encouraging, their applicability is restricted, and it would be advantageous to collect data from a broader range of populations in terms of ethnicity and geographical location. Lastly, more information regarding VitD supplementation, dietary intake, and sunlight exposure after birth are also contributors to after-birth VitD status, which is equally important for child growth after birth.

## 5. Conclusions

Maternal vitamin D status in three trimesters throughout pregnancy may not have a persistent effect on the offspring’s dynamic growth trajectory. However, higher vitamin D status in the third trimester and the cord blood is associated with a reduced risk of the WAZ high-rising trajectory from birth to 4 years of age, signifying a lower likelihood of obesity later in life. Further research is necessary to explore the long-term association between maternal vitamin D concentrations and high-rising WAZ outcomes, as well as the most effective period for vitamin D intervention during pregnancy.

## Figures and Tables

**Table 1:** Distribution of vitamin D status and maternal–child characteristics selected for analysis in Shanghai Birth Cohort, Shanghai, China, recruited from 2013 to 2016.

Characteristics	Overall (959)	LAZ (n = 950)	WAZ (n = 959)	HCZ (n = 895)	WLZ (n = 948)
	n (%)	n (%)	n (%)	n (%)	n (%)
**Serum 25(OH)D (ng/mL)**					
25(OH)D at T1 (Mean ± SD)	26.16 ± 10.20	26.18 ± 10.18	26.16 ± 10.20	25.96 ± 10.13	26.15 ± 10.18
25(OH)D at T2 (Mean ± SD)	31.93 ± 11.37	31.91 ± 11.37	31.93 ± 11.37	31.74 ± 11.37	31.88 ± 11.35
25(OH)D at T3 (Mean ± SD)	35.75 ± 13.33	35.78 ± 13.32	35.75 ± 13.33	35.59 ± 13.33	35.75 ± 13.31
25(OH)D in CB (Mean ± SD)	19.87 ± 9.07	19.91 ± 9.09	19.87 ± 9.07	19.85 ± 9.09	19.90 ± 9.10
25(OH)D categories at T1 (ng/mL)					
<20	291 (30.86)	288 (30.84)	291 (30.86)	277 (31.51)	288 (30.90)
≥20, <30	309 (32.77)	305 (32.66)	309 (32.77)	289 (32.88)	305 (32.73)
≥30	343 (36.37)	341 (36.51)	343 (36.37)	313 (35.61)	339 (36.37)
25(OH)D categories at T2 (ng/mL)					
<20	142 (16.38)	141 (16.41)	142 (16.38)	137 (16.91)	141 (16.45)
≥20, <30	251 (28.95)	249 (28.99)	251 (28.95)	236 (29.14)	249 (29.05)
≥30	474 (54.67)	469 (54.60)	474 (54.67)	437 (53.95)	467 (54.49)
25(OH)D categories at T3 (ng/mL)					
<20	112 (11.99)	111 (11.97)	112 (11.99)	106 (12.17)	111 (12.00)
≥20, <30	218 (23.34)	214 (23.09)	218 (23.34)	204 (23.42)	214 (23.14)
≥30	604 (64.67)	602 (64.94)	604 (64.67)	561 (64.41)	600 (64.86)
25(OH)D categories in CB (ng/mL)					
<12	182 (18.98)	180 (18.95)	182 (18.98)	173 (19.35)	180 (18.99)
≥12, <20	370 (38.58)	365 (38.42)	370 (38.58)	343 (38.37)	365 (38.50)
≥20	407 (42.44)	405 (42.63)	407 (42.44)	378 (42.28)	403 (42.51)
**Maternal characteristics**					
Maternal age (y)					
<25	76 (8.08)	76 (8.14)	76 (8.08)	74 (8.42)	76 (8.15)
≥25, <30	520 (55.26)	515 (55.14)	520 (55.26)	488 (55.52)	513 (55.04)
≥30, <35	289 (30.71)	287 (30.73)	289 (30.71)	264 (30.03)	287 (30.79)
≥35	56 (5.95)	56 (6.00)	56 (5.95)	53 (6.03)	56 (6.01)
Maternal educational level					
Below high school	71 (7.57)	70 (7.52)	71 (7.57)	67 (7.65)	70 (7.53)
High school	207 (22.07)	207 (22.23)	207 (22.07)	194 (22.15)	206 (22.17)
College	524 (55.86)	520 (55.85)	524 (55.86)	491 (56.05)	519 (55.87)
Bachelor’s degree or above	136 (14.50)	134 (14.39)	136 (14.50)	124 (14.16)	134 (14.42)
Prepregnancy BMI (kg/m^2^)					
<18.5	141 (15.02)	141 (15.13)	141 (15.02)	136 (15.51)	141 (15.16)
≥18.5, <25	716 (76.25)	709 (76.07)	716 (76.25)	664 (75.71)	707 (76.02)
≥25	82 (8.73)	82 (8.80)	82 (8.73)	77 (8.78)	82 (8.82)
Gestational weight gain (kg)					
<12	386 (42.60)	383 (42.51)	386 (42.60)	362 (42.69)	382 (42.49)
≥12, <15	289 (31.90)	288 (31.96)	289 (31.90)	268 (31.60)	288 (32.04)
≥15, <18	155 (17.11)	155 (17.20)	155 (17.11)	146 (17.22)	155 (17.24)
≥18	76 (8.39)	75 (8.32)	76 (8.39)	72 (8.49)	74 (8.23)
GDM					
No	754 (84.34)	749 (84.35)	754 (84.34)	703 (84.09)	747 (84.31)
Yes	140 (15.66)	139 (15.65)	140 (15.66)	133 (15.91)	139 (15.69)
**Child characteristics**					
Child gender					
Male	496 (51.72)	492 (51.79)	496 (51.72)	465 (52.01)	491 (51.79)
Female	463 (48.28)	458 (48.21)	463 (48.28)	429 (47.99)	457 (48.21)
Delivery mode					
Natural delivery	553 (60.44)	547 (60.24)	553 (60.44)	514 (60.12)	545 (60.15)
Cesarean delivery	362 (39.56)	361 (39.76)	362 (39.56)	341 (39.88)	361 (39.85)
Breastfeeding duration					
<6 months	147 (25.70)	146 (25.66)	147 (25.70)	139 (25.32)	146 (25.70)
≥6 months	425 (74.30)	423 (74.34)	425 (74.30)	410 (74.68)	422 (74.30)

WAZ, weight-for-age z-score; LAZ, length-for-age z-score; HCZ, head circumference-for-age z-score WLZ, weight-for-length z-score; BMI, body mass index; T1, early pregnancy; T2, middle pregnancy; T3, late pregnancy; CB, cord blood; GDM, gestational diabetes mellitus.

**Table 2:** The persistent effect of maternal vitamin D status throughout pregnancy on growth trajectories was analyzed with the generalized estimating equation (GEE).

Growth Trajectories	25(OH)D, 10 ng/mL	
	OR (95% CI)	*p*
**Weight-for-age z-score (WAZ)**		
Low-stable (n = 220)	Reference	
Moderate-increasing (n = 543)	1.04 (0.94,1.15)	0.82
High-increasing (n = 197)	0.87 (0.76,0.99)	0.08
**Length-for-age z-score (LAZ)**		
Low-stable (n = 402)	Reference	
Low-decreasing (n = 133)	1.03 (0.91,1.17)	1.00
Moderate-increasing (n = 340)	0.94 (0.86, 1.03)	0.60
High-increasing (n = 75)	1.07 (0.93, 1.24)	1.00
**Head circumference-for-age z-score (HCZ)**		
Moderate-increasing (n = 552)	Reference	
Low-stable (n = 232)	0.99 (0.90, 1.09)	1.00
High-increasing (n = 111)	1.00 (0.88, 1.14)	1.00
**Weight-for-length z-score (WLZ)**		
Low-increasing (n = 331)	Reference	
Moderate-increasing (n = 560)	0.95 (0.87, 1.04)	0.50
High-increasing vs. (n = 57)	1.00 (0.93, 1.07)	1.00

WAZ, weight-for-age z-score; LAZ, length-for-age z-score; HCZ, head circumference-for-age z-score WLZ, weight-for-length z-score. *p* was adjusted with Bonferroni correction.

**Table 3:** Odds ratio (OR) and 95% confidence interval (CI) for trajectory groups in each anthropometrical measure according to per 10 ng/mL increase in 25(OH)D level (continuous variables).

Trajectory Groups	T1, 10 ng/mL		T2, 10 ng/mL		T3, 10 ng/mL		CB, 10 ng/mL	
	Adjusted OR (95% CI)	*p*	Adjusted OR (95% CI)	*p*	Adjusted OR (95% CI)	*p*	Adjusted OR (95% CI)	*p*
**WAZ**								
Low-stable (n = 220)	Reference		Reference		Reference		Reference	
Moderate-increasing (n = 543)	1.06 (0.90, 1.25)	0.84	1.01 (0.87, 1.17)	1.00	1.05 (0.92, 1.20)	0.90	0.82 (0.69, 0.98)	0.06
High-increasing (n = 197)	1.01 (0.81, 1.26)	1.00	0.91 (0.74, 1.12)	0.74	0.75 (0.62, 0.91)	<0.01	0.57 (0.43, 0.76)	<0.01
**LAZ**								
Low-stable (n = 402)	Reference		Reference		Reference		Reference	
Low-decreasing (n = 133)	1.04 (0.85, 1.28)	1.00	1.04 (0.86, 1.25)	1.00	1.10 (0.95, 1.28)	0.63	1.15 (0.93, 1.42)	0.63
Moderate-increasing (n = 340)	0.98 (0.84, 1.15)	1.00	0.95 (0.82, 1.11)	1.00	0.94 (0.83, 1.06)	0.96	0.81 (0.67, 0.96)	0.06
High-increasing vs. low-stable (n = 75 vs. n = 402)	1.20 (0.94, 1.54)	0.42	1.07 (0.83, 1.37)	1.00	1.01 (0.81, 1.25)	1.00	0.84 (0.60, 1.16)	0.84
**HCZ**								
Moderate-increasing (n = 552)	Reference		Reference		Reference		Reference	
Low-stable (n = 232)	0.94 (0.80, 1.11)	1.00	1.03 (0.88, 1.20)	1.00	0.96 (0.85, 1.09)	1.00	1.08 (0.91, 1.28)	0.80
High-increasing (n = 111)	0.96 (0.60, 1.53)	1.00	0.74 (0.47, 1.16)	0.38	1.02 (0.64, 1.63)	1.00	1.21 (0.77, 1.90)	0.80
**WLZ**								
Low-increasing (n = 331)	Reference		Reference		Reference		Reference	
Moderate-increasing (n = 560)	0.99 (0.85, 1.14)	1.00	1.00 (0.87, 1.14)	1.00	0.93 (0.83, 1.05)	0.48	0.85 (0.72, 1.00)	0.08
High-increasing (n = 57)	1.10 (0.81, 1.48)	1.00	0.73 (0.53, 1.00)	0.10	0.77 (0.59, 1.01)	0.12	0.64 (0.42, 0.98)	0.08

Adjusted maternal age, maternal education, pre-pregnancy BMI, gestational weight gain, delivery mode, and child sex. WAZ, weight-for-age z-score; LAZ, length-for-age z-score; HCZ, head circumference-for-age z-score WLZ, weight-for-length z-score; T1, early pregnancy; T2, middle pregnancy; T3, late pregnancy; CB, cord blood. *p* was adjusted with Bonferroni correction.

## Data Availability

The data presented in this study are available on request from the corresponding author. The data are not publicly available because they contain information that could compromise the privacy of research participants.

## Supplementary Materials

The following supporting information can be downloaded at: https://www.mdpi.com/article/10.3390/nu16152410/s1, Table S1: Bayesian information criterion (BIC) and 2∆BIC for model selection in group-based trajectory model (GBTM); Table S2: Polynomial power for model selection in group-based trajectory model (GBTM); Table S3: Anthropometric measures in children at birth, 42 days, 6 months, 12 months, 24 months and 48 months; Table S4: The number of participants in each trajectory; Table S5: Odds ratio (OR) and 95% confidence interval (CI) for trajectory groups in each anthropometrical measure according to per 10ng/mL increase in average 25(OH)D level throughout pregnancy; Table S6: Odds ratio (OR) and 95% confidence interval (CI) for trajectory groups of weight-for-age length-for-age, weight-for-length and head-circumference-for-age according to 25(OH)D levels (categorical variables); Table S7: Adjusted Odds ratio (OR) and 95% confidence interval (CI) for trajectory groups according to 25(OH)D levels (stratified by gender); Table S8: Adjusted Odds ratio (OR) and 95% confidence interval (CI) for trajectory groups according to 25(OH)D levels (stratified by maternal age).

## Abbreviations

VitD, vitamin D; CB, cord blood; WAZ, weight-for-age z-score; LAZ, length-for-age z-score; HCZ, head circumference-for-age z-score; WLZ, weight-for-length z-score; GBTM, group-based trajectory model.

## References

[1] Giustina A., Adler R.A., Binkley N., Bollerslev J., Bouillon R., Dawson-Hughes B., Ebeling P.R., Feldman D., Formenti A.M., Lazaretti-Castro M. (2020). Consensus statement from 2(nd) International Conference on Controversies in Vitamin D. Rev. Endocr. Metab. Disord..

[2] Bennour I., Haroun N., Sicard F., Mounien L., Landrier J.F. (2022). Recent insights into vitamin D, adipocyte, and adipose tissue biology. Obes. Rev..

[3] Samuel S., Sitrin M.D. (2008). Vitamin D’s role in cell proliferation and differentiation. Nutr. Rev..

[4] Mulligan M.L., Felton S.K., Riek A.E., Bernal-Mizrachi C. (2010). Implications of vitamin D deficiency in pregnancy and lactation. Am. J. Obstet. Gynecol..

[5] Wagner C.L., Hollis B.W. (2018). The Implications of Vitamin D Status During Pregnancy on Mother and her Developing Child. Front. Endocrinol..

[6] Zhao R., Zhou L., Wang S., Yin H., Yang X., Hao L. (2022). Effect of maternal vitamin D status on risk of adverse birth outcomes: A systematic review and dose-response meta-analysis of observational studies. Eur. J. Nutr..

[7] Larsen S.D., Christensen M.E., Dalgard C., Lykkedegn S., Andersen L.B., Andersen M.S., Glintborg D., Christesen H.T. (2020). Pregnancy or cord 25-hydroxyvitamin D is not associated with measures of body fat or adiposity in children from three months to three years of age. An Odense Child Cohort study. Clin. Nutr..

[8] Razaghi M., Gharibeh N., Vanstone C.A., Sotunde O.F., Wei S.Q., McNally D., Rauch F., Jones G., Weiler H.A. (2022). Maternal excess adiposity and serum 25-hydroxyvitamin D < 50 nmol/L are associated with elevated whole body fat mass in healthy breastfed neonates. BMC Pregnancy Childbirth.

[9] Daraki V., Roumeliotaki T., Chalkiadaki G., Katrinaki M., Karachaliou M., Leventakou V., Vafeiadi M., Sarri K., Vassilaki M., Papavasiliou S. (2018). Low maternal vitamin D status in pregnancy increases the risk of childhood obesity. Pediatr. Obes..

[10] Jiang X., Lu J., Zhang Y., Teng H., Pei J., Zhang C., Guo B., Yin J. (2021). Association between maternal vitamin D status with pregnancy outcomes and offspring growth in a population of Wuxi, China. Asia Pac. J. Clin. Nutr..

[11] Drozdz D., Alvarez-Pitti J., Wojcik M., Borghi C., Gabbianelli R., Mazur A., Herceg-Cavrak V., Lopez-Valcarcel B.G., Brzezinski M., Lurbe E. (2021). Obesity and Cardiometabolic Risk Factors: From Childhood to Adulthood. Nutrients.

[12] Hou Y., Wang M., Yang L., Zhao M., Yan Y., Xi B. (2019). Weight status change from childhood to early adulthood and the risk of adult hypertension. J. Hypertens..

[13] Luo J., Hodge A., Hendryx M., Byles J.E. (2020). Age of obesity onset, cumulative obesity exposure over early adulthood and risk of type 2 diabetes. Diabetologia.

[14] Giles L.C., Whitrow M.J., Davies M.J., Davies C.E., Rumbold A.R., Moore V.M. (2015). Growth trajectories in early childhood, their relationship with antenatal and postnatal factors, and development of obesity by age 9 years: Results from an Australian birth cohort study. Int. J. Obes..

[15] Liu J.X., Liu J.H., Frongillo E.A., Boghossian N.S., Cai B., Hazlett L.J. (2017). Body mass index trajectories during infancy and pediatric obesity at 6 years. Ann. Epidemiol..

[16] Correia-Branco A., Keating E., Martel F. (2015). Maternal undernutrition and fetal developmental programming of obesity: The glucocorticoid connection. Reprod. Sci..

[17] Hauta-Alus H.H., Kajantie E., Holmlund-Suila E.M., Rosendahl J., Valkama S.M., Enlund-Cerullo M., Helve O.M., Hytinantti T.K., Viljakainen H., Andersson S. (2019). High Pregnancy, Cord Blood, and Infant Vitamin D Concentrations May Predict Slower Infant Growth. J. Clin. Endocrinol. Metab..

[18] Eckhardt C.L., Gernand A.D., Roth D.E., Bodnar L.M. (2015). Maternal vitamin D status and infant anthropometry in a US multi-centre cohort study. Ann. Hum. Biol..

[19] Crozier S.R., Harvey N.C., Inskip H.M., Godfrey K.M., Cooper C., Robinson S.M., Group S.W.S.S. (2012). Maternal vitamin D status in pregnancy is associated with adiposity in the offspring: Findings from the Southampton Women’s Survey. Am. J. Clin. Nutr..

[20] Francis E.C., Hinkle S.N., Song Y., Rawal S., Donnelly S.R., Zhu Y., Chen L., Zhang C. (2018). Longitudinal Maternal Vitamin D Status during Pregnancy Is Associated with Neonatal Anthropometric Measures. Nutrients.

[21] Zhang J., Tian Y., Wang W., Ouyang F., Xu J., Yu X., Luo Z., Jiang F., Huang H., Shen X. (2019). Cohort profile: The Shanghai Birth Cohort. Int. J. Epidemiol..

[22] Jiao X., Yuan Y., Wang X., Li J., Liu B., Yuan T., Yu X. (2020). Development of a sensitive HPLC-MS/MS method for 25-hydroxyvitamin D2 and D3 measurement in capillary blood. J. Clin. Lab. Anal..

[23] Ross A.C., Taylor C.L., Yaktine A.L., Del Valle H.B. (2011). Dietary Reference Intakes for Calcium and Vitamin D.

[24] Holick M.F., Binkley N.C., Bischoff-Ferrari H.A., Gordon C.M., Hanley D.A., Heaney R.P., Murad M.H., Weaver C.M., Endocrine S. (2011). Evaluation, treatment, and prevention of vitamin D deficiency: An Endocrine Society clinical practice guideline. J. Clin. Endocrinol. Metab..

[25] Kiely M., O’Donovan S.M., Kenny L.C., Hourihane J.O., Irvine A.D., Murray D.M. (2017). Vitamin D metabolite concentrations in umbilical cord blood serum and associations with clinical characteristics in a large prospective mother-infant cohort in Ireland. J. Steroid Biochem. Mol. Biol..

[26] Wang X., Jiao X., Tian Y., Zhang J., Zhang Y., Li J., Yang F., Xu M., Yu X., Shanghai Birth Cohort S. (2021). Associations between maternal vitamin D status during three trimesters and cord blood 25(OH)D concentrations in newborns: A prospective Shanghai birth cohort study. Eur. J. Nutr..

[27] World Health Organization, WHO Multicentre Growth Reference Study Group (2006). WHO Child Growth Standards: Length/Height-for-Age, Weight-for-Age, Weight-for-Length, Weight-for-Height and Body Mass Index-for-Age: Methods and Development.

[28] Nagin D.S., Jones B.L., Passos V.L., Tremblay R.E. (2018). Group-based multi-trajectory modeling. Stat. Methods Med. Res..

[29] Song M. (2019). Trajectory analysis in obesity epidemiology: A promising life course approach. Curr. Opin. Endocr. Metab. Res..

[30] Amberntsson A., Barebring L., Winkvist A., Lissner L., Meltzer H.M., Brantsaeter A.L., Papadopoulou E., Augustin H. (2022). Maternal vitamin D status in relation to infant BMI growth trajectories up to 2 years of age in two prospective pregnancy cohorts. Obes. Sci. Pract..

[31] Hyde N.K., Brennan-Olsen S.L., Wark J.D., Hosking S.M., Holloway-Kew K.L., Pasco J.A. (2018). Vitamin D during pregnancy and offspring body composition: A prospective cohort study. Pediatr. Obes..

[32] Wong R.S., Tung K.T.S., Mak R.T.W., Leung W.C., Yam J.C., Chua G.T., Fung G.P.G., Ho M.H.K., Wong I.C.K., Ip P. (2022). Vitamin D concentrations during pregnancy and in cord blood: A systematic review and meta-analysis. Nutr. Rev..

[33] The American College of Obstetricians and Gynecologists Prenatal Development: How the Baby Grows during Pregnancy. https://www.acog.org/womens-health/faqs/how-your-fetus-grows-during-pregnancy.

[34] Renault K.M., Carlsen E.M., Norgaard K., Nilas L., Pryds O., Secher N.J., Cortes D., Jensen J.E., Olsen S.F., Halldorsson T.I. (2015). Intake of carbohydrates during pregnancy in obese women is associated with fat mass in the newborn offspring. Am. J. Clin. Nutr..

[35] Brei C., Stecher L., Meyer D.M., Young V., Much D., Brunner S., Hauner H. (2018). Impact of Dietary Macronutrient Intake during Early and Late Gestation on Offspring Body Composition at Birth, 1, 3, and 5 Years of Age. Nutrients.

[36] Noviandhari A., Faisal F., Dhamayanti M. (2022). Correlation of Maternal Prenatal Vitamin D Level with Postnatal Infant Growth in Length and Head Circumference: A Cohort Study on Vitamin D Status and Its Impact During Pregnancy and Childhood in Indonesia. Int. J. Gen. Med..

[37] Gould J.F., Anderson A.J., Yelland L.N., Smithers L.G., Skeaff C.M., Zhou S.J., Gibson R.A., Makrides M. (2017). Association of cord blood vitamin D with early childhood growth and neurodevelopment. J. Paediatr. Child Health.

[38] Maugeri A., Barchitta M., Blanco I., Agodi A. (2019). Effects of Vitamin D Supplementation During Pregnancy on Birth Size: A Systematic Review and Meta-Analysis of Randomized Controlled Trials. Nutrients.

[39] Hauta-Alus H.H., Holmlund-Suila E.M., Kajantie E., Rosendahl J., Valkama S.M., Enlund-Cerullo M., Andersson S., Makitie O. (2021). The Effects of Vitamin D Supplementation During Infancy on Growth During the First 2 Years of Life. J. Clin. Endocrinol. Metab..

[40] Hu Y., Wang R., Mao D., Chen J., Li M., Li W., Yang Y., Zhao L., Zhang J., Piao J. (2021). Vitamin D Nutritional Status of Chinese Pregnant Women, Comparing the Chinese National Nutrition Surveillance (CNHS) 2015–2017 with CNHS 2010–2012. Nutrients.

