# Supplementary material for: Associations between Longitudinal Maternal and Cord Blood Vitamin D Status and Child Growth Trajectories Up to 4 Years of Age
Source: Nutrients. 2024 Jul 25;16(15):2410. doi: 10.3390/nu16152410 (PMC11313987; doi:10.3390/nu16152410)
Supplement: Supplementary file 1 [file nutrients-16-02410-s001.zip › nutrients-3087704-supplementary.pdf]

Table S1. Bayesian information criterion (BIC) and 2∆BIC for model selection in group-based trajectory model (GBTM).

Number of groups	BIC	Null model	2∆BIC
Weight-for-age z-score (WAZ)			
1	-6404.84		
2	-5913.04	1	983.60
3	-5794.80	2	236.48
4	-5753.05	3	83.5
5	-5722.94	4	60.22
Length-for-age z-score (LAZ)			
1	-6319.02		
2	-5789.18	1	1059.68
3	-5682.11	2	214.14
4	-5656.59	3	51.04
5	-5636.83	4	39.52
Head-circumference-for-age z-score (HCZ)			
1	-5848.14		
2	-5508.15	1	679.98
3	-5388.24	2	239.82
4	-5374.04	3	28.40
5	-5369.06	4	9.96
Weight-for-length z-score (WLZ)			
1	-6895.65		
2	-6680.37	1	430.56
3	-6651.69	2	57.36
4	-6651.29	3	0.80
5	-6649.16	4	4.26

Table S2. Polynomial power for model selection in group-based trajectory model (GBTM).

Polynomial	BIC	Null model	2ΔBIC
Weight-for-age z-score (WAZ)			
1	-5928.02		
2	-5794.80	1	266.44
3	-5696.10	2	197.4
Length-for-age z-score (LAZ)			
1	-5710.55		
2	-5656.59	1	107.92
3	-5602.72	2	107.74
Head-circumference-for-age z-score (HCZ)			
1	-5380.05		
2	-5388.24	1	-16.38
3	-5392.14	2	-7.8
Weight-for-length z-score (WLZ)			
1	-6760.14		
2	-6651.69	1	216.90
3	-6572.22	2	158.94

Table S3. Anthropometric measures in children at birth, 42 days, 6 months, 12 months, 24 months and 48 months.

	At birth	42 days	6 months	12 months	24 months	48 months
**Weight**						
N	934	890	777	792	854	532
Original value(kg)	3.41 ± 0.41	5.19 ± 0.58	8.63 ± 1.05	10.32 ± 1.14	12.93 ± 1.46	18.03 ± 2.93
z-score	0.22 ± 0.84	0.62 ± 0.84	0.94 ± 1.00	0.79 ± 0.89	0.64 ± 0.89	0.25 ± 1.03
**Length/Height**						
N	830	865	779	792	854	536
Original value(cm)	49.94 ± 1.10	56.90 ± 2.03	68.79 ± 2.51	76.31 ± 2.57	88.76 ± 3.15	108.73 ± 4.81
z-score	0.22 ± 0.60	0.62 ± 1.00	0.81 ± 1.03	0.43 ± 0.9	0.60 ± 0.96	0.43 ± 0.98
**Head circumference**						
N	812	853	765	779	754	397
Original value(cm)	34.45 ± 1.18	37.84 ± 1.17	43.50 ± 1.32	45.82 ± 1.37	48.20 ± 1.39	50.60 ± 1.47
z-score	0.22 ± 0.96	0.13 ± 0.96	0.47 ± 0.94	0.19 ± 0.97	0.31 ± 0.95	0.35 ± 0.98
**Weight-for-length**						
N	829	860	783	790	853	512
z-score	0.17 ± 1.11	0.21 ± 1.20	0.75 ± 1.04	0.80 ± 0.96	0.40 ± 0.97	-0.07 ± 1.12

Table S4. The number of participants in each trajectory.

Trajectory groups	Total number of participants n (%)
**Weight-for-age z-score (WAZ)**	
Low-stable	220 (22.94)
Moderate-increasing	543 (56.52)
High-increasing	197 (20.54)
**Length-for-age z-score (LAZ)**	
Low-decreasing	133 (14.00)
Low-stable	402 (42.32)
Moderate-increasing	340 (35.79)
High-increasing	75 (7.89)
**Head-circumference-for-age z-score (HCZ)**	
Low-stable	232 (25.92)
Moderate-increasing	552 (61.68)
High-increasing	111 (12.40)
**Weight-for-length z-score (WLZ)**	
Low-increasing	331 (34.92)
Moderate-increasing	560 (59.07)
High-increasing	57 (6.01)

Table S5. Odds ratio (OR) and 95% confidence interval (CI) for trajectory groups in each anthropometrical measure according to per 10ng/mL increase in average 25(OH)D level throughout pregnancy.

Trajectory groups	Adjusted OR (95%CI)	*P*
**Weight-for-age z-score (WAZ)**		
Low-stable (n=220)	Reference	
Moderate-increasing (n=543)	1.01 (0.99, 1.02)	0.81
High-increasing (n=197)	0.82 (0.64, 1.04)	0.20
**Length-for-age z-score (LAZ)**		
Low-stable (n=402)	Reference	
Low-decreasing (n=133)	1.14 (0.92, 1.41)	0.63
Moderate-increasing (n=340)	0.95 (0.80, 1.12)	1.00
High-increasing (n=75)	1.16 (0.88, 1.53)	0.87
**Head-circumference-for-age z-score (HCZ)**		
Moderate-increasing (n=552)	Reference	
Low-stable (n=232)	0.98 (0.82, 1.16)	1.00
High-increasing (n=111)	1.05 (0.83, 1.33)	1.00
**Weight-for-length z-score (WLZ)**		
Low-increasing (n=331)	Reference	
Moderate-increasing (n=560)	0.95 (0.82, 1.11)	1.00
High-increasing (n=57)	0.75 (0.53, 1.06)	0.22

Ave VitD, average 25(OH)D levels during three trimesters. P was adjusted with Bonferroni correction.

Table S6. Odds ratio (OR) and 95% confidence interval (CI) for trajectory groups of weight-for-age length-for-age, weight-for-length and head-circumference-for-age according to 25(OH)D levels (categorical variables).

Trajectory groups	Adjusted OR (95% CI)											
	**T1** (ng/mL)			**T2** (ng/mL)			**T3** (ng/mL)			**CB** (ng/mL)		
	≥30 (n= 343)	<30 (n= 600)	*P*	≥30 (n= 474)	<30 (n=393)	*P*	≥30 (n=604)	<30 (n=330)	*P*	≥20 (n=407)	<20 (n=552)	*P*
**WAZ**												
Low-stable (n=220)		Reference			Reference			Reference			Reference	
Moderate-increasing (n=543)	1.0	0.96 (0.68, 1.35)	1.00	1.0	1.12 (0.79, 1.59)	1.00	1.0	0.99 (0.69, 1.44)	1.00	1.0	1.40 (1.00, 1.96)	0.10
High-increasing (n=197)	1.0	1.24 (0.77, 1.98)	0.76	1.0	1.16 (0.72, 1.86)	1.00	1.0	2.04 (1.27, 3.26)	0.006	1.0	2.58 (1.60, 4.16)	<0.01
**LAZ**												
Low-stable (n=402)		Reference			Reference			Reference			Reference	
Low-decreasing (n=133)	1.0	0.83 (0.54, 1.27)	1.00	1.0	0.79 (0.51, 1.22)	0.87	1.0	0.87 (0.55, 1.38)	1.00	1.0	0.82 (0.54, 1.24)	1.00
Moderate-increasing (n=340)	1.0	1.02 (0.73, 1.42)	1.00	1.0	1.01 (0.72, 1.40)	1.00	1.0	1.23 (0.88, 1.71)	0.69	1.0	1.56 (1.13, 2.14)	<0.01
High-increasing (n=75)	1.0	0.62 (0.36, 1.07)	0.27	1.0	0.93 (0.53, 1.63)	1.00	1.0	1.08 (0.61, 1.91)	1.00	1.0	1.08 (0.63, 1.84)	0.78
**HCZ**												
Moderate-increasing (n=552)		Reference			Reference			Reference			Reference	
Low-stable (n=232)	1.0	1.00 (0.71, 1.41)	1.00	1.0	0.84 (0.59, 1.18)	0.62	1.0	1.00 (0.71, 1.42)	1.00	1.0	0.83 (0.60, 1.15)	0.26
High-increasing (n=111)	1.0	0.96 (0.60, 1.53)	1.00	1.0	0.74 (0.47, 1.16)	0.38	1.0	1.02 (0.64, 1.63)	1.00	1.0	1.21 (0.77, 1.90)	0.08
**WLZ**												
Low-increasing (n=331)		Reference			Reference			Reference			Reference	
Moderate-increasing (n=56)	1.0	1.07 (0.79, 1.45)	1.00	1.0	0.96 (0.71, 1.31)	1.00	1.0	1.16 (0.85, 1.60)	0.70	1.0	1.50 (1.12, 2.02)	0.02
High-increasing (n=57)	1.0	0.85 (0.44, 1.66)	1.00	1.0	1.53 (0.78, 2.99)	0.44	1.0	2.97 (1.51, 5.82)	<0.01	1.0	1.38 (0.73, 2.64)	0.64

Adjusted maternal age, maternal education, pre-pregnancy BMI, gestational weight gain, delivery mode and child sex. WAZ, weight-for-age z-score; LAZ, length-for-age z-score; HCZ, head-circumference-for-age z-score WLZ, weight-for-length z-score; T1, early pregnancy; T2, middle pregnancy; T3, late pregnancy; CB, cord blood. P was adjusted with Bonferroni correction.

Table S7. Adjusted Odds ratio (OR) and 95% confidence interval (CI) for trajectory groups according to 25(OH)D levels (stratified by gender).

Trajectory groups	T1, 10ng/mL		T2, 10ng/mL		T3, 10ng/mL		CB, 10ng/mL	
	adjusted OR (95% CI)	*P*	adjusted OR (95% CI)	*P*	adjusted OR (95% CI)	*P*	adjusted OR (95% CI)	*P*
**Male**								
**WAZ**								
Low-stable (n=220)	Reference		Reference		Reference		Reference	
Moderate-increasing (n=543)	1.06 (0.85, 1.33)	1.00	0.87 (0.71, 1.08)	0.42	0.92 (0.77, 1.09)	0.66	0.76 (0.60, 0.96)	0.04
High-increasing (n=197)	0.98 (0.73, 1.32)	1.00	0.79 (0.59, 1.06)	0.24	0.67 (0.50, 0.88)	0.01	0.46 (0.30, 0.70)	< 0.01
**LAZ**								
Low-stable (n=402)	Reference		Reference		Reference		Reference	
Low-decreasing (n=133)	0.96 (0.75, 1.24)	1.00	0.94 (0.74, 1.18)	1.00	1.09 (0.90, 1.33)	1.00	1.13 (0.88, 1.46)	1.00
Moderate-increasing (n=340)	0.85 (0.69, 1.04)	0.33	0.80 (0.66, 0.97)	0.09	0.86 (0.74, 1.02)	0.24	0.73 (0.58, 0.93)	0.03
High-increasing (n=75)	1.17 (0.85, 1.61)	0.96	1.05 (0.76, 1.44)	1.00	1.05 (0.80, 1.36)	1.00	0.73 (0.47, 1.13)	0.45
**HCZ**								
Moderate-increasing (n=552)								
Low-stable (n=232)	0.91 (0.73, 1.13)	0.80	1.04 (0.88, 1.23)	1.00	1.11 (0.89, 1.39)	0.70	1.01 (0.80, 1.27)	1.00
High-increasing (n=111)	1.05 (0.77, 1.45)	1.00	0.93 (0.67, 1.29)	1.00	0.84 (0.64, 1.12)	0.48	0.58 (0.37, 0.90)	0.04
**WLZ**								
Low-increasing (n=331)	Reference		Reference		Reference		Reference	
Moderate-increasing (n=56)	0.92 (0.75, 1.13)	0.90	0.99 (0.82, 1.19)	1.00	1.07 (0.91, 1.26)	0.80	1.17 (0.94, 1.45)	0.30
High-increasing (n=57)	1.13 (0.79, 1.62)	1.00	0.95 (0.64, 1.41)	1.00	0.84 (0.61, 1.15)	0.56	0.74 (0.47, 1.18)	0.40
**Female**								
**WAZ**								
Low-stable (n=220)	Reference		Reference		Reference		Reference	
Moderate-increasing (n=543)	1.11 (0.87, 1.42)	0.80	1.23 (0.98, 1.55)	0.16	1.26 (1.03, 1.54)	0.06	0.91 (0.69, 1.19)	0.98
High-increasing (n=197)	1.03 (0.71, 1.48)	1.00	1.05 (0.76, 1.44)	1.00	0.85 (0.64, 1.13)	0.54	0.73 (0.48, 1.11)	0.28
**LAZ**								
Low-stable (n=402)	Reference		Reference		Reference		Reference	
Low-decreasing (n=133)	1.17 (0.86, 1.59)	0.93	1.14 (0.88, 1.50)	0.64	1.06 (0.85, 1.31)	1.00	1.14 (0.82, 1.58)	1.00
Moderate-increasing (n=340)	1.11 (0.90, 1.37)	0.99	1.05 (0.87, 1.26)	1.00	0.96 (0.81, 1.13)	1.00	0.82 (0.65, 1.05)	0.33
High-increasing (n=75)	1.25 (0.89, 1.76)	0.57	1.11 (0.81, 1.51)	1.00	0.90 (0.67, 1.21)	1.00	0.88 (0.58, 1.34)	1.00
**HCZ**								
Moderate-increasing (n=552)	Reference		Reference		Reference		Reference	
Low-stable (n=232)	0.98 (0.75, 1.28)	1.00	0.85 (0.69, 1.04)	0.22	1.04 (0.78, 1.39)	1.00	0.89 (0.67, 1.18)	0.84
High-increasing (n=111)	1.09 (0.80, 1.50)	1.00	1.36 (1.03, 1.80)	0.06	1.04 (0.82, 1.33)	1.00	1.20 (0.83, 1.73)	0.68
**WLZ**								
Low-increasing (n=331)	Reference		Reference		Reference		Reference	
Moderate-increasing (n=56)	1.08 (0.87, 1.34)	0.98	0.99 (0.81, 1.21)	1.00	1.07 (0.89, 1.27)	0.96	1.23 (0.96, 1.58)	0.18
High-increasing (n=57)	0.92 (0.56, 1.49)	1.00	0.51 (0.30, 0.86)	0.02	0.86 (0.63, 1.18)	0.72	1.16 (0.60, 2.23)	1.00

Adjusted for maternal age, pre-pregnancy BMI, pregnancy weigh gain, gestational week.T1, early pregnancy; T2, middle pregnancy; T3, late pregnancy; CB, cord blood. P was adjusted with Bonferroni correction.

Table S8. Adjusted Odds ratio (OR) and 95% confidence interval (CI) for trajectory groups according to 25(OH)D levels (stratified by maternal age).

**Trajectory groups**	**T1, 10ng/mL**		**T2, 10ng/mL**		**T3, 10ng/mL**		**CB, 10ng/mL**	
	**adjusted OR (95% CI)**	***P***	**adjusted OR (95% CI)**	***P***	**adjusted OR (95% CI)**	***P***	**adjusted OR (95% CI)**	***P***
**Maternal age <30**								
**WAZ**								
Low-stable (n=220)	Reference		Reference		Reference		Reference	
Moderate-increasing (n=543)	1.10 (0.90, 1.35)	0.68	0.97 (0.80, 1.18)	1.00	1.05 (0.89, 1.23)	1.00	0.87 (0.69, 1.08)	0.40
High-increasing (n=197)	0.97 (0.72, 1.32)	1.00	0.87 (0.67, 1.14)	0.74	0.83 (0.65, 1.06)	0.26	0.61 (0.42, 0.89)	0.02
**LAZ**								
Low-stable (n=402)								
Low-decreasing (n=133)	0.95 (0.73, 1.22)	1.00	1.12 (0.89, 1.41)	0.99	1.14 (0.96, 1.37)	0.42	1.09 (0.85, 1.42)	1.00
Moderate-increasing (n=340)	0.90 (0.75, 1.08)	0.81	0.91 (0.77, 1.08)	0.84	0.89 (0.77, 1.03)	0.39	0.71 (0.57, 0.88)	<0.01
High-increasing (n=75)	0.99 (0.72, 1.35)	1.00	0.99 (0.74, 1.32)	1.00	1.07 (0.82, 1.38)	1.00	0.78 (0.53, 1.16)	0.69
**HCZ**								
Moderate-increasing (n=552)								
Low-stable (n=232)	0.98 (0.80, 1.19)	1.00	1.07 (0.88, 1.30)	1.00	0.99 (0.85, 1.15)	1.00	1.07 (0.86, 1.32)	1.00
High-increasing (n=111)	0.85 (0.63, 1.16)	0.62	1.13 (0.84, 1.51)	0.84	0.94 (0.73, 1.20)	1.00	0.92 (0.65, 1.31)	1.00
**WLZ**								
Low-increasing (n=331)								
Moderate-increasing vs. (n=560)	1.02 (0.86, 1.22)	1.00	1.04 (0.88, 1.24)	1.00	1.05 (0.91, 1.22)	1.00	1.26 (1.03, 1.54)	0.04
High-increasing (n=57)	1.03 (0.71, 1.49)	1.00	0.79 (0.54, 1.16)	0.46	0.96 (0.74, 1.25)	1.00	0.94 (0.60, 1.46)	1.00
**Maternal age ≥30**								
**WAZ**								
Low-stable (n=220)								
Moderate-increasing (n=543)	0.96 (0.72, 1.28)	1.00	1.05 (0.81, 1.35)	1.00	1.07 (0.85, 1.34)	1.00	0.73 (0.54, 0.99)	0.08
High-increasing (n=197)	1.00 (0.71, 1.41)	1.00	0.93 (0.66, 1.30)	1.00	0.63 (0.44, 0.89)	< 0.01	0.54 (0.34, 0.83)	< 0.01
**LAZ**								
Low-stable (n=402)								
Low-decreasing (n=133)	1.19 (0.87, 1.63)	0.84	0.88 (0.67, 1.17)	1.00	0.95 (0.74, 1.22)	1.00	1.17 (0.85, 1.62)	1.00
Moderate-increasing (n=340)	1.08 (0.85, 1.39)	1.00	0.94 (0.76, 1.17)	1.00	0.97 (0.80, 1.17)	1.00	0.92 (0.70, 1.21)	1.00
High-increasing (n=75)	1.50 (1.05, 2.14)	0.06	1.18 (0.83, 1.69)	1.00	0.86 (0.63, 1.17)	0.99	0.82 (0.52, 1.30)	1.00
**HCZ**								
Moderate-increasing (n=552)								
Low-stable (n=232)	0.87 (0.64, 1.17)	0.70	0.97 (0.75, 1.26)	1.00	0.88 (0.70, 1.11)	0.56	1.08 (0.80, 1.47)	1.00
High-increasing (n=111)	1.28 (0.94, 1.75)	0.24	1.16 (0.86, 1.56)	0.96	1.00 (0.77, 1.30)	1.00	0.86 (0.59, 1.26)	0.88
**WLZ**								
Low-increasing (n=331)								
Moderate-increasing vs. (n=560)	1.03 (0.81, 1.33)	1.00	0.97 (0.78, 1.21)	1.00	1.12 (0.93, 1.37)	0.54	1.08 (0.83, 1.40)	1.00
High-increasing (n=57)	1.22 (0.74, 2.01)	0.88	0.69 (0.42, 1.16)	0.32	0.78 (0.51, 1.21)	0.54	0.80 (0.45, 1.39)	0.84

Adjusted for gender, pre-pregnancy BMI, pregnancy weigh gain, gestational week. T1, early pregnancy; T2, middle pregnancy; T3, late pregnancy; CB, cord blood; Ave VitD, average 25(OH)D levels during three trimesters. P was adjusted with Bonferroni correction.
